# Global Perspectives on Rabies Control and Elimination: A Scoping Review of Dog Owners’ Knowledge, Attitudes, and Practices

**DOI:** 10.3390/pathogens14080728

**Published:** 2025-07-23

**Authors:** Moumita Das, Valeriia Yustyniuk, Andres M. Perez, Maria Sol Perez Aguirreburualde

**Affiliations:** 1 Center for Animal Health and Food Safety, College of Veterinary Medicine, University of Minnesota, Saint Paul, MN 55108, USA; yusty002@umn.edu (V.Y.); aperez@umn.edu (A.M.P.); mperezag@umn.edu (M.S.P.A.); 2 Department of Veterinary Population Medicine, University of Minnesota, Saint Paul, MN 55108, USA

**Keywords:** rabies, dogs, vaccine-preventable disease, knowledge, attitudes, practice

## Abstract

Rabies is a fatal but entirely vaccine-preventable disease, with the highest risk in areas where free-roaming domestic dogs are prevalent. Understanding dog owners’ knowledge, attitudes, and practices (KAP) is crucial for shaping effective rabies control strategies. This scoping review aimed to synthesize global evidence from studies evaluating dog owners’ KAP to identify behavioral factors relevant to rabies prevention and control. A systematic literature search was conducted using PubMed, Web of Science, and Scopus, covering the period from 2012 to 2025. Seventy full-text articles were included based on predefined criteria. The findings reveal substantial gaps in dog owners’ knowledge, beliefs, and behaviors regarding rabies prevention. While general awareness of rabies is high among dog owners, their knowledge about transmission, clinical signs, and the fatal nature of the disease is inconsistent, with significant variability across studies. The vaccination uptake also varied widely across studies, ranging from less than 1% to over 90%, with no study reporting full coverage. Furthermore, a strong positive correlation was found between vaccination practice and the awareness of vaccine benefits (r = 0.69, *p* = 0.004). Common barriers to vaccination include lack of information, vaccine accessibility, distance to clinics, and personal constraints. These insights underscore the importance of early and targeted communication about vaccination campaigns. Future research should focus on periodically evaluating KAP before and after interventions to better inform rabies control efforts.

## 1. Introduction

Rabies is a viral zoonotic disease caused by the rabies virus of the *Lyssavirus* genus within the Rhabdoviridae family [1]. The disease remains endemic in more than 150 countries, placing nearly half of the global population at risk of exposure [2]. Rabies is a neglected tropical disease prioritized by the World Health Organization (WHO) due to its disproportionate impact on underserved populations, with an estimated 59,000 human deaths annually, mainly in Africa and Asia [3,4].

While all warm-blooded mammals are susceptible to rabies, dogs remain the primary reservoir and source of infection in most low- and middle-income countries [5]. More than 99% of the human rabies deaths are caused by bites from infected domestic dogs, making dog-mediated transmission the principal route of infection globally [6,7]. In rabies-endemic areas, large populations of unvaccinated, free-roaming dogs serve as a persistent reservoir, maintaining the cycle of transmission. Dog bites are the single greatest risk factor for human exposure, and children under 15 years of age account for approximately 40% of all rabies victims [8]. Sub-Saharan Africa bears one of the highest burdens of dog-mediated rabies, where dog bite injuries are common and access to timely and affordable post-exposure prophylaxis (PEP) remains inadequate [9]. The economic cost of dog-mediated rabies is also substantial, with over USD 500 million spent annually on PEP alone, not including indirect costs such as travel, lost income, and long-term psychological effects on bite victims [10].

Rabies is considered to be one of the best-known zoonotic diseases, with substantial efforts dedicated to its control [5]. Although the disease is highly fatal and presents distinctive signs—features that have been central to awareness campaigns across regions—it remains underreported in many parts of the world. Underreporting is often attributed to weak surveillance systems, limited coverage of veterinary services, poor health-seeking behavior, logistical barriers to healthcare and diagnosis, and the limited use of differential diagnosis, especially in fatal cases that may be misclassified as other diseases, such as malaria in endemic areas [11,12]. In parallel, access to life-saving PEP, crucial for preventing the onset of symptoms after exposure in humans, is often hindered by high costs, stockouts, geographical barriers, and lack of awareness [13].

Dog-mediated human rabies is an example of a One Health challenge involving close interaction between humans and animals [5]. Both humans and animals are under the same disease threat, and the control effort requires substantial and systematic coordination across sectors. Controlling disease transmission from dogs to humans through vaccination not only reduces the reliance on PEP but has also been proven to be a cost-effective strategy, lowering the overall expenses associated with rabies prevention and control efforts [14]. Despite being an ultimately vaccine-preventable disease, rabies still causes thousands of deaths every year, especially in low- and middle-income countries [15]. One significant barrier to effective control is the lack of coordination between the veterinary and public health sectors [16]. In many settings, disagreement and misalignment over whether rabies control should be led by the health or agriculture ministry continues to delay the development and implementation of effective national strategies. The separation between veterinary and public health responsibilities undermines the initiatives of integrated responses and limits the ability to rapidly and effectively contain outbreaks [17].

Despite these challenges, rabies control and elimination are achievable. Rabies in dogs has been successfully eliminated in many high-income countries and regions, including Canada, the United States, Western Europe, Japan, and Australia. Their success largely stems from sustained mass dog vaccination campaigns initiated in the early 20th century, along with robust public education, dog population management, and legal enforcement of responsible dog ownership [18,19,20,21]. Notably, Latin American countries, supported by the Pan American Health Organization (PAHO), initiated regional control efforts in the 1980s, leading to a 98% reduction in canine rabies cases [22,23,24]. These achievements underscore the importance of a strong government commitment, effective technical coordination, and community involvement. On the other hand, many countries where canine rabies is endemic remain in the early stages of their control efforts. These territories often face persistent barriers, including weak disease surveillance systems, a partial understanding of local rabies epidemiology, inadequate cold chain infrastructure, a high prevalence of ownerless and free-roaming dogs, limited funding, and poor public awareness [5,25]. These factors contribute to the consistently low vaccination coverage, undermining rabies eradication goals. Achieving and maintaining at least 70% vaccination coverage in the dog population, a key threshold for rabies elimination, requires strong intersectoral collaboration, effective community engagement, responsible dog ownership, and sustainable dog population management [25,26].

In this context, understanding how people perceive rabies disease and acting upon that knowledge are essential for designing effective preventive measures. Knowledge, attitudes, and practices (KAP) studies are effective tools for evaluating public awareness, beliefs, and behaviors relating to health risks and interventions [27]. Regarding rabies, a KAP study will help us identify the drivers influencing dog owners’ decisions to vaccinate their pets or engage in risky behaviors, highlight gaps in public knowledge, and reveal social and cultural barriers that impact vaccination uptake and disease reporting [28]. The nature and scope of KAP studies on rabies are extensive due to the wide range of host species, study populations, locations, and socio-economic status across various countries. Therefore, this scoping review aims to systematically identify and synthesize global evidence on dog owners’ knowledge, attitudes, and practices related to rabies.

Although the KAP articles reviewed in this study were taken from across the globe, the majority originated from Africa and Asia, the regions that bear the highest rabies burden worldwide. These geographic concentrations reflect both the increased prevalence of the disease in these areas [29] and a growing research interest in evaluating public knowledge and behaviors as a basis for effective control strategies. With the varied knowledge, attitudes, and practices concerning rabies among dog owners, this scoping review focuses on how people’s knowledge and attitudes influence their practices. By synthesizing global evidence, this review aims to identify patterns, uncover gaps, and suggest improvements in rabies prevention strategies, emphasizing the critical role of dog owners in controlling the disease.

## 2. Methodology

### 2.1. Establishing Research Questions

This scoping review was guided by a list of research questions broadly categorized into two focal areas: basic understanding of rabies, and the implementation of control measures by dog owners. Within the basic understanding category, four key questions were addressed: whether dog owners had heard of rabies, whether they knew that dogs could act as reservoirs for rabies, their recognition of clinical signs, and their understanding that rabies is fatal once symptoms appear. The second category, related to rabies control measures, was divided into two subthemes: the uptake of dog vaccination, and responsible dog ownership (RDO). Questions related to dog vaccination investigated owners’ knowledge about the importance of vaccination, the appropriate age for puppies to receive their first dose, and the necessity of annual boosters. Attitudes toward vaccinating pets were summarized under both paid and free-of-charge scenarios. Finally, the information about dog vaccination practices was synthesized by determining whether owners had vaccinated their dogs and whether they maintained vaccination records. Additionally, reasons for not vaccinating the dogs were also extracted. The RDO component included questions exploring knowledge, attitudes, and practices regarding dog confinement or leashing, sterilization, provision of food and shelter, dog registration and licensing, and access to regular veterinary care.

### 2.2. Search and Selection of Articles

**Figure 1:**
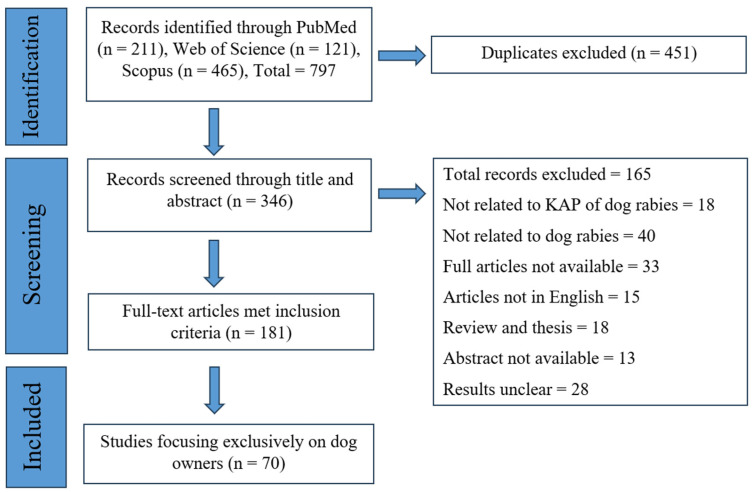
PRISMA flow diagram.

A comprehensive literature search was carried out using three electronic databases: PubMed, Web of Science, and Scopus. The keywords used in the investigation were dog rabies, canine rabies, prevention, control, knowledge, awareness, attitudes, perception, and practice. The search strategy followed the Preferred Reporting Items for Systematic Reviews and Meta-Analyses (PRISMA) guidelines for conducting and reporting this systematic review (Figure 1). Customized search techniques were employed for each database, considering their unique indexing words, to ensure thorough coverage of appropriate information. These search techniques combined phrases, including MeSH (Medical Subject Headings) terms and “Boolean” operators (and/or). The following general keywords were included in the search string for the database: ((KAP OR Knowledge OR Attitude* OR practice*) AND (dog* OR canine*) AND rabies). All selected articles focused on dog owners’ knowledge, attitudes, perceptions, and behaviors regarding rabies control and prevention and were published between January 2012 and March 2025.

### 2.3. Setting up Inclusion and Exclusion Criteria

All studies satisfying the following criteria were included: (1) KAP studies inquiring about the prevention and eradication of canine rabies; (2) studies that included both dog owners and community members who provided food and shelter to domestic dogs; (3) studies written and published in English; (4) geographic region: global; and (5) studies conducted between January 2012, and March 2025. Only original, peer-reviewed research articles were considered for inclusion, with the majority employing a cross-sectional study design, typically through surveys or structured interviews, to assess KAP related to rabies in dog owners. Articles were excluded if they (1) focused on rabies in species other than dogs (such as bats, squirrels, raccoons, or wild animals); (2) lacked defined KAP-related outcomes or presented findings without clear descriptions, metrics, or thematic interpretations relevant to rabies prevention; (3) were abstracts, commentaries, anonymous reports, letters, or editorials; (4) focused on KAP related to human rabies without connection to dog ownership; (5) covered other public health issues despite satisfying KAP design; (6) involved laboratory-based vaccine or molecular experiments; or (7) were review articles.

### 2.4. Quality Assessment

To assess the methodological quality of the included studies, we used a modified version of the critical appraisal tool by Downes and Brennan [30], systematically evaluating key components, including aims, methods, results, discussion, and limitations. Special emphasis was placed on the meticulous development and execution of surveys, the precise interpretation of results, and the comprehensive delineation of constraints. The accessibility of raw data, including provisions for sharing, played a crucial role in affirming the research’s transparency. This extensive evaluation facilitated nuanced insights into the articles’ strengths and limitations. The total quality scores, aligned with the study objectives, ranged from 0 to 10, categorizing studies into low (1–4), moderate (5–7), or high (8–10) quality.

### 2.5. Data Extraction and Reporting of Results

Two researchers independently extracted the data from the selected articles, and a third researcher resolved any contradictions. Descriptive data from each article were recorded on a Google spreadsheet, including author(s), year of publication, sample size, study outcome, country and continent, and journal of publication. The extracted data focused on the respondents’ basic understanding of rabies and vaccines, their attitudes toward vaccination under both paid and free-of-charge scenarios, and vaccination-related practices, such as whether they vaccinated their dogs and kept records. Additional data were collected on KAP related to responsible dog ownership, including practices such as dog confinement or leashing, sterilization, provision of basic care (food and shelter), registration and licensing, and use of veterinary services.

### 2.6. Correlation Analysis

This study examined the relationship of dog vaccination practices with three other factors: the belief that rabies vaccination is beneficial, awareness of rabies as a fatal disease, and whether dog owners permit their dogs to roam freely. These variables were selected because they are commonly reported across many studies, along with vaccination rates. Spearman’s rank correlation test was used to measure the strength and direction of the relationships between each pair of variables. This non-parametric test was chosen because the data came from a small number of studies, might not follow a normal distribution, and included potential outliers [31]. For each study included, vaccination practice was defined as the proportion of surveyed dog owners who reported vaccinating their dogs. Belief in the benefit of vaccination was defined as the proportion who agreed that vaccinating dogs helps prevent rabies. Awareness of rabies fatality was the proportion of owners who correctly recognized that rabies leads to death once clinical signs appear. Roaming behavior described the proportion of owners who reported allowing their dogs to roam freely outside the home.

## 3. Results

Out of the 181 articles that met the inclusion criteria, 70 specifically focused on dog owners’ knowledge, attitudes, and practices (KAP) related to rabies prevention in dogs. These 70 studies formed the primary basis for data extraction, thematic synthesis, and correlation analysis in this review.

**Figure 2:**
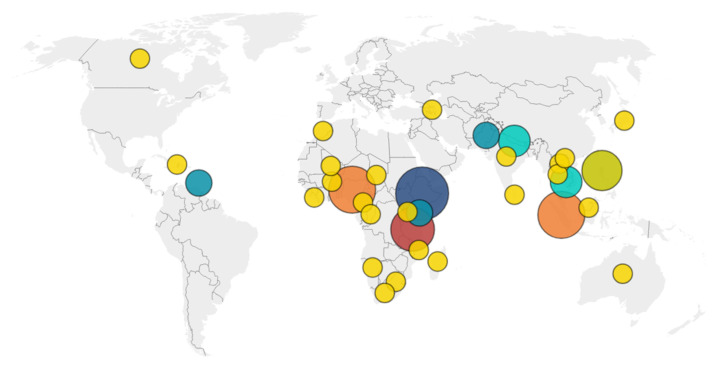
Geographical distribution of reviewed articles. The size of the circles is proportional to the number of articles evaluated, which is also indicated by their color (yellow = 1, blue = 2, light blue = 3, green = 5, red = 6, orange = 7, dark blue = 9).

Table S1 summarizes key details of the reviewed articles, including the publication year, sample size, study location, and journal source. Figure 2 represents the geographic distribution of the studies: 54% were conducted in Africa ( 38 articles), 39% in Asia ( 27 articles), 6% in the Americas (4 articles), and 1% in Oceania (1 article). The unit of analysis varied across the studies; some focused on individual dog owners, while others targeted households, typically represented by interviews with a single respondent per household.

### 3.1. Basic Understanding of Rabies

**Figure 3:**
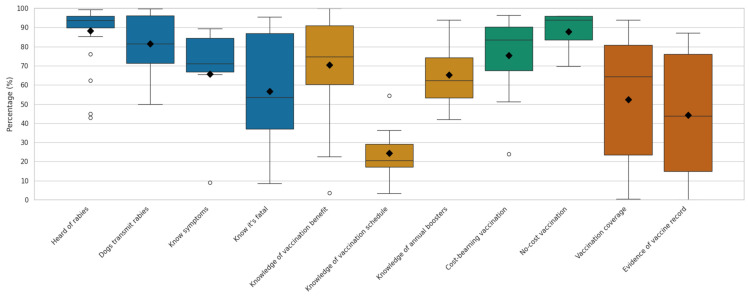
Boxplots show the distribution of responses across subcategories related to basic understanding of rabies (blue), dog vaccination awareness (yellow), vaccination attitudes under cost scenarios (green), and reported practices (orange). The black diamond indicates the mean value for each subcategory, while the box represents the interquartile range (IQR), with the median shown as the horizontal line within the box. The whiskers indicate variability outside the upper and lower quartiles.

Four key areas were identified from the selected articles to synthesize the overall knowledge of dog owners in managing dog rabies: (a) respondents have heard about rabies, (b) respondents know that dogs serve as a reservoir for rabies, (c) they can identify clinical signs of rabies and, and (d) they know that rabies is fatal when clinical signs appear. Among the 70 reviewed articles, 26 studies specifically reported on participants’ awareness of rabies (Table S2). Of these, 19 studies found that over 90% of respondents had heard of rabies, and 3 studies reported awareness levels between 80% and 90%; in comparison, the remaining 4 studies recorded lower awareness levels, ranging from 45% to 65%. As shown in the box-and-whisker plots in Figure 3 (blue bars), the latter three knowledge parameters showed more significant variability in responses compared to general awareness.

Eighteen studies addressed respondents’ knowledge of dogs being a rabies reservoir. The lowest proportion, 50%, was reported by Jama and Mengistu [32], who stated that participants were aware of the transmission of rabies through both dog bites and infected saliva. In contrast, a study performed in Tanzania reported that 99.76% of respondents correctly identified dogs as a source of rabies transmission. Recognition of clinical signs of rabies in dogs was reported in 10% of the studies (7/70). Among them, Edukugho et al. recorded the highest recognition rate, at 88.8% in Nigeria [33], while another Nigerian study by Ijoma et al. reported that only 9% of participants could describe clinical features of rabies in dogs [34]. Lastly, the awareness of rabies being fatal was addressed in 29% (20/70) of the articles, with a wide variability in the responses, ranging from 8.6% to 95.5%. Notably, a survey from Indonesia found that despite 90.9% of respondents having heard of rabies, only 8.6% were aware of its fatality once clinical signs appeared [35].

In our study, conducted in Turkana, a group of veterinary professionals, rather than dog owners, was engaged to assess the community’s knowledge, attitudes, and practices regarding canine rabies [36]. Approximately 42.4% of the survey respondents reported that fewer than half of the community members had heard of rabies. A larger group, 75.8%, felt that most Turkana people could not recognize the clinical signs of rabies in dogs. Additionally, 60.6% believed that less than half of the residents knew that rabies could spread from dogs to humans, and 63.6% assumed that few people understood that rabies is fatal once symptoms initiate.

### 3.2. Vaccination of Dogs

We examined three key aspects of dog owners’ understanding of canine rabies vaccination: the benefits of rabies vaccination, awareness of the vaccination schedule, and the necessity of annual boosters. Twenty-nine studies reported respondents’ awareness of the benefits of rabies vaccination, with the results ranging from 3.59% to 100%. In addition, seven articles addressed participants’ knowledge about the vaccination schedule in dogs, and five articles discussed the awareness of the need for annual booster doses (Table S2).

In Turkana County, Kenya, the surveyed veterinary professionals reported that most people in the community lacked an understanding of the importance of vaccinating dogs against rabies, with over 80% stating that less than half of the population recognized its usefulness. Approximately 63.6% believed that only a small number of dog owners ensured that their dogs received annual rabies shots. However, interest in vaccination increased when the vaccine was offered for free; 51.5% of respondents said that more than half of the community would be willing to vaccinate under such conditions. Still, nearly 90% of professionals confirmed that the overall vaccination rate stayed below 50% [36].

Attitudes toward canine rabies vaccination varied based on whether the service was free or at cost. Eleven studies reported dog owners’ willingness to pay for rabies vaccination, with responses ranging from 24% to 96.5%. Five of these studies also assessed willingness to vaccinate if the vaccine were provided free of cost. In those five studies, willingness was notably high, ranging from 69.7% to 96% (Table S2).

Overall, 46 out of 70 reviewed studies (65%) reported the implementation of routine canine rabies vaccination based on self-reported information from dog owners. Two studies, from Cambodia and Chad, showed extremely low vaccination coverage of 0.8% and 0.5%, respectively [37,38]. In contrast, two studies from India [39] and Nigeria [33] reported coverage rates exceeding 90%. None of the studies reported 100% vaccination coverage. Significantly, administering vaccinations did not always correlate with record-keeping (vaccination certificates). This review identified 12 articles that mentioned vaccination records, with evidence ranging from 0% to 87% (Table S2). The plot shows a clear positive association (*p*-value = 0.002) between vaccination coverage and the availability of vaccination records (Figure S1). The studies with higher reported vaccination rates also tended to show a greater presence of documented evidence. In Figure 3, the orange bars display the distribution of values from 12 studies that reported both the proportion of dog owners who vaccinated their dogs and the proportion who could provide a vaccination certificate or other proof of vaccination. The median values and distribution ranges for the two indicators appear broadly similar, suggesting that, in studies with higher vaccination rates, documented evidence of vaccination was also relatively more common.

### 3.3. Correlation Between Vaccination Practices and Related Variables

Spearman’s correlation coefficients were calculated to explore the association between dog vaccination practices and three key variables: the belief in the usefulness of rabies vaccination, awareness of the fatal nature of rabies, and whether owners permit their dogs to roam outside. These variables were selected primarily because a considerable number of articles reported these specific indicators, allowing for a reasonable sample size to perform the correlation analysis. Additionally, these variables are relevant due to their potential influence on responsible dog ownership and vaccination behavior. The first two variables reflect the owner’s knowledge and perceived importance of canine vaccination, while the third characterizes a behavioral practice that may indicate general attitudes toward animal care. A statistically significant (*p*-value = 0.004) and positive correlation (Figure S2) was observed between vaccination practices and knowledge about the benefits of vaccination, suggesting that individuals who recognize the role of immunization in preventing rabies are more likely to vaccinate their dogs (Table 1). A weaker, yet still positive, correlation was found between vaccination practices and awareness of rabies fatality, indicating that owners who recognize the deadly nature of the disease are more persuaded to vaccinate their dogs (Figure S3). Although the correlation was weak, the data suggest that dog owners who allow their pets to wander freely are less likely to engage in vaccination (Figure S4).

### 3.4. Barriers to Canine Rabies Vaccination

To examine the underlying reasons for non-vaccination of dogs, all of the reviewed publications were analyzed, and the findings were categorized into four major themes (Table S3). The most frequently reported reasons included knowledge-related barriers or misperceptions, cited by twelve studies, either about rabies disease itself, ongoing vaccination campaigns, or concerns about disease transmission during these campaigns. Barriers related to cost and service availability, also reported in twelve studies, included limited vaccine availability, shortages of veterinarians or vaccine providers, concerns over vaccine side effects, and a lack of trust in vaccine effectiveness. Logistical challenges such as long distances to veterinary clinics and lack of transportation were documented in seven articles. In addition, some articles mentioned owner-related personal factors, such as negligence, inability to restrain or handle dogs, the young age of the dogs, dogs being away during the campaign, recent relocation, or even reluctance to disclose specific reasons.

### 3.5. Responsible Dog Ownership (RDO)

Table S2 summarizes the findings on responsible dog ownership, categorized into three spheres: knowledge, attitudes, and practices. The owners recognized the need for leashing in three studies conducted within the knowledge domain. These studies were conducted in two distinct regions of the Philippines. The two studies from Pampanga Province reported an awareness rate of over 80% [40,41], while the study from Panglao Island showed a considerably lower level of knowledge, at 36% [42]. In contrast, only one study reported a 96.8% awareness of providing dogs with food, water, and shelter to prevent them from scavenging [41]. Knowledge about dog registration and licensing was documented in five articles, with both the highest (90.7%) and lowest (39%) responses coming from the Philippines [40,42]. Additionally, regular veterinary care for pets was mentioned in two articles.

Under the attitudes, five articles emphasized a positive attitude toward keeping dogs on leashes, with the responses ranging from 65.3% [41] to 98.2% [43]. Three studies indicated that owners believed that sterilization or neutering could contribute to rabies control. One study reported that 60% of respondents expressed a positive attitude toward the importance of providing food, water, and shelter to their pets [44]. Additionally, three studies highlighted favorable attitudes toward dog registration and licensing, with the reported rates varying widely from 20.1% in Ethiopia [45] to 96.2% in Mozambique [46].

In practice, 13 studies reported keeping dogs on leashes, while 24 documented that owners allowed their dogs to wander freely. Sterilization or neutering practices were mentioned in eight studies, and the provision of adequate food, water, and shelter was reported in ten. Dog registration and licensing were practiced in only two studies, and periodic medical care was observed in seven.

## 4. Discussion

Rabies remains a public health threat in many parts of the world, predominantly in settings where human and animal health systems face structural and resource-related challenges [47]. This scoping review aimed to synthesize evidence related to dog owners’ knowledge, attitudes, and practices (KAP) in relation to rabies prevention and control. KAP studies are an essential tool to identify gaps between awareness, beliefs, and behaviors [48]. We observed a wide variability in KAP outcomes from 70 selected studies in the current review. Understanding these contextual factors is critical for designing culturally appropriate and sustainable rabies control strategies in regions with high dog populations and limited access to veterinary services. Addressing rabies effectively depends not only on technical solutions, such as vaccination, but also on community behavioral changes, as highlighted by KAP assessments that show the link between awareness and improved dog care [49].

The synthesis of four key knowledge indicators suggests that most respondents have at least heard of rabies, likely due to exposure through mass media, community programs, or personal experiences. In contrast, the other three indicators reflect a decline in more specific and potentially actionable knowledge, such as understanding transmission routes or recognizing clinical signs. While general awareness is high, fewer respondents understand that dogs are the primary reservoir of the virus. Similarly, knowledge of clinical signs and the fatal nature of the disease is associated with lower median values and greater variability. The wide range of knowledge regarding rabies fatality emphasizes inconsistency in knowledge dissemination, possibly due to differences in educational outreach, health communication strategies, or cultural perceptions across regions. One study included in this review reported that although 62% of respondents had heard of rabies, only 24% of them expressed concern about its fatality [50]. Similarly, a study conducted in the Philippines found that 85% of participants were aware of rabies, but many lacked an understanding of the specific transmission mechanisms between animals and humans [51]. This disparity between general awareness about rabies and detailed knowledge is concerning. The findings underscore the need for educational strategies that extend beyond raising awareness. Some studies suggest that academic achievement may play a role in promoting rabies awareness. For example, individuals with secondary education or higher were reported to be more likely to be informed about rabies than those with only primary education in specific study settings [35,40]. This pattern may help explain the variability in knowledge levels across study populations; however, further research is needed to confirm its consistency across different regions. Moreover, children often have frequent and direct contact with dogs; therefore, these findings support the recommendation to include rabies education in elementary school curricula [52].

In addition to general awareness of the disease, the knowledge about rabies vaccination varied considerably across studies. Understanding the value of vaccination had the highest median and mean scores, with relatively low variability, suggesting general agreement among dog owners about its importance. However, knowledge about the vaccination schedule and annual boosters was much lower and showed broader variability. Very few studies assessed all three aspects together. For example, in Rwanda, although more than 80% of the community acknowledged the importance of rabies vaccination, only 20.6% knew the recommended age for a puppy’s first dose [53]. No information is available regarding this knowledge gap in this study. However, this inconsistency highlights that while general information about the value of vaccination has reached many communities, detailed knowledge of vaccination protocols remains limited. The potential reasons for this might include inadequate health communication or veterinary engagement between the national and grassroots levels.

Eleven studies reported dog owners’ willingness to vaccinate their animals if they had to pay for the vaccine, while only five of these also explored willingness under free-of-charge scenarios. A study conducted in Thailand found that socio-economic status influenced the desire to pay for rabies vaccination, with individuals from lower-income backgrounds exhibiting more positive attitudes toward immunization. Participants were allocated into three clusters based on their willingness to pay: high, moderate, and low, with average amounts of nearly USD 7.00, USD 2.53, and USD 1.07, respectively [54]. Although it might seem intuitive to state that those unwilling to pay might vaccinate if it were free, such assumptions cannot be established without the paired individual-level responses. The limited number of studies that reported both scenarios makes it difficult to draw a conclusion about the effect of cost on vaccination behavior. This inconsistency represents a limitation in how some studies were designed and highlights the need for more consistent and detailed surveys to better understand owners’ motivations in different financial situations.

A study conducted in Tanzania reported that most responders were willing to pay for vaccines, but not more than USD 0.31 [55]. In Chad, vaccination coverage dropped to under 25% when owners were obligated to pay, compared to 64–87% during earlier free vaccination campaigns [56]. These findings suggest that while dog owners recognize the value of vaccination, financial barriers can limit actual uptake. In another example, a study by Hasanov et al. asked participants if they would vaccinate their dogs in both free and paid scenarios. In both cases, 6 out of 100 people said “No” [57]. If these six were the same individuals, it reflects that there is always a small group who are not willing to vaccinate their dogs, regardless of the cost. This result suggests that cost alone is not the only barrier; additional behavioral and motivational factors must also be addressed.

The positive association between vaccination practices and dog owners’ understanding of rabies underscores the importance of targeted education and awareness campaigns. Moreover, studies revealed that owners who understood the fatal nature of rabies or the utility of vaccination were more likely to participate in vaccination campaigns. These findings are supported by an Ethiopian study that revealed a significant association between vaccination intention and dog owners’ knowledge of rabies [58]. Dog owners who regularly vaccinate their dogs tend to restrict their dogs’ free movement. This perception could be because such owners are generally more responsible or informed, practicing confinement and preventive care [59]. In contrast, owners who allow their dogs to roam freely may not see themselves as fully accountable for their care or may face practical barriers, such as difficulty capturing the animal.

Among the articles presenting the reasons for non-vaccination, eight studies reported that the lack of proper information regarding vaccination campaigns was a common barrier [38,46,53,60,61,62,63,64]. This may result from inadequate pre-campaign advertisement or outreach limitations in remote communities, where residents often learn about the campaign on the actual day that it occurs. It is recommended that communities be reached through commonly used media and social platforms, such as television, radio, newspapers, and public gatherings that are culturally relevant in the particular area [53], tailored to the cultural context, and delivered in local languages, with visual aids to enhance understanding [65,66].

Logistical challenges also played a significant role. In some settings, long distances to veterinary clinics and challenges in handling dogs during campaigns were often cited as major barriers [38,53,61,63,67,68]. These could potentially be addressed through central-point or door-to-door vaccination strategies [69] and the oral rabies vaccination (ORV) approach, recently endorsed by the Food and Agriculture Organization (FAO), World Health Organization (WHO), and World Organization for Animal Health (WOAH) [70]. Further restrictions reported included misconceptions about the appropriate age for vaccination and skepticism about vaccine efficacy. In many communities, there is limited awareness of the WHO’s guidance to vaccinate puppies under three months of age, including newborns, in rabies-endemic regions [53]. Remarkably, more than half of the articles reviewed in the current study identified that dog owners often failed to vaccinate their animals due to informational and behavioral factors rather than physical inaccessibility, suggesting that many barriers could be mitigated through well-targeted education and engagement efforts.

The concept of responsible dog ownership (RDO) aims to address problems associated with stray dogs, and to improve animal welfare [71]. RDO entails providing food, shelter, medical care, social interaction, and opportunities for natural behavior, as well as ensuring legal responsibilities like immunization [72]. Twenty-four publications reported the presence of free-roaming domestic dogs, often allowed to wander freely outside during the day and return home at night. Rural settings, lower socio-economic status, and certain religious and cultural norms often contribute to this practice [46,73]. In densely populated areas, such free-roaming dogs are more likely to come into contact with potentially rabid animals, increasing the risk of transmission [74].

High rates of dog reproduction contribute to the presence of abandoned puppies, many of which lack adequate care. These animals are often left without sufficient food, shelter, or veterinary care, increasing their vulnerability and the risk of disease transmission [75]. Ten of the reviewed studies reported on whether owners provided basic care—food, water, and shelter—for their dogs. Providing food regularly to dogs is particularly important, because hungry animals may roam, increasing the likelihood of contact with potentially rabid animals [76]. In Addis Ababa, for example, over 70% of study participants reported not providing medical care to their dogs, with the misconception that rabies is the only disease that can ever affect dogs, and that the anti-rabies vaccine is sufficient for that [77].

The quality assessment of the included articles in the present study revealed that approximately 80% of them fell within the moderate (5–7)-to-high (8–10) quality range, indicating generally sound methodological standards across all of these studies. Most of the articles clearly articulated their research objectives and identified appropriate target populations, contributing to higher scores in these domains. However, lower scores were attributed to limitations in the methodology and interpretation of results, including unclear sample size and target population, inconsistent human–dog ratios, imprecise definitions, and indistinct inclusion criteria [37,77,78,79] An observed issue concerned the presentation of numerical data without accompanying contextual information, which sometimes led to unsupported attempts at data extrapolation to broader populations. Additionally, instances were noted where authors omitted the provision of raw data and the survey questionnaire, thereby limiting the reader’s ability to assess the validity of the conclusions and forcing reliance on the authors’ interpretations [63,77,80,81,82,83] These omissions introduce ambiguity and raise concerns about the reproducibility and robustness of the findings.

Inadequate documentation of key methodological components, particularly the development, validation, and administration of survey tools, further contributes to uncertainty about the overall reliability of results. These limitations not only affect the internal validity of individual studies but also hinder the collective utility of this body of research to inform evidence-based interventions and policy updates. In the context of rabies control, where timely and targeted public health strategies are essential, weak methodological transparency can reduce the credibility and applicability of study findings for designing effective vaccination campaigns, educational outreach, and dog population management programs.

## 5. Strengths and Limitations

This scoping review serves as a valuable resource for guiding future research and informing the design of interventions and evidence-based policy decisions in the context of rabies elimination. It is likely the first comprehensive global review focused specifically on dog owners’ KAP related to rabies management. The findings reveal substantial gaps between what participants know, believe, and practice—insights that can support the development of more targeted and effective intervention strategies. Correlation analyses further illustrate patterns of association between knowledge, attitudes, and subsequent practices, reinforcing the importance of integrated behavioral frameworks in rabies control.

A structured quality assessment was conducted using defined criteria to evaluate the methodological rigor of the included studies. However, this review also presents certain limitations. By focusing on a 14-year period, relevant studies published before this timeframe may have been excluded. Additionally, the literature search was restricted to three scientific databases, which may have led to the omission of pertinent publications available elsewhere. The KAP parameters selected for inclusion were defined by the objectives of this review, yet other aspects of rabies control, such as dog population dynamics, human–animal interaction patterns, or broader One Health considerations, may also offer important insights.

However, considerable heterogeneity in research design, methodology, and data reporting across studies complicated the synthesis of the findings and limited the generalizability of the results. These challenges were particularly evident in the correlation analysis, where the use of secondary data from diverse studies, varying in quality and scope, may affect the robustness and interpretability of observed associations. This underscores the need for more standardized and methodologically rigorous approaches in future KAP research on rabies.

## 6. Recommendations and Future Directions

To improve rabies control, targeted educational campaigns should focus on high-risk populations, particularly children and rural dog owners, using community-based and school-centered approaches. Expanding access to dog vaccination through mobile clinics and community-led initiatives can help reach underserved areas and increase coverage. Integrating a One Health approach with coordination between the veterinary and public health sectors is essential for sustainable progress. Future research should prioritize longitudinal KAP studies, standardized methodologies, and the inclusion of behavioral and ecological factors such as dog population management. Embedding KAP assessments into national rabies programs will support real-time strategy adjustments and progress monitoring.

## 7. Conclusions

This review reveals persistent gaps in dog owners’ knowledge, attitudes, and practices related to rabies prevention. While general awareness is high, behavioral and logistical barriers continue to limit effective control. Strengthening educational efforts, improving access to vaccination, and promoting responsible dog ownership, especially through community engagement and veterinary leadership, are essential. To make KAP studies more relevant for policy and programming, there is a critical need to improve their research quality, design, and consistency. These studies should not only inform strategies but also guide pilot interventions (such as cost-sharing vaccination models) that can support sustainability and reduce long-term dependence on external donors. Regular integration of KAP assessments into rabies control programs is vital to monitor progress and adjust efforts accordingly.

## Figures and Tables

**Table 1:** Spearman’s correlation between dog vaccination practice and three selected variables: belief that vaccination is useful, awareness that rabies is fatal, and whether owners allow their dogs to roam freely.

Parameter 1	Parameter 2	No. of Articles Matched	Correlation Coefficients	*p*-Value
Practice of vaccination	Vaccination is useful	15	0.69	0.004
	Rabies is fatal	11	0.25	0.467
	Allowed to roam	17	−0.36	0.159

## Supplementary Materials

The following supporting information can be downloaded at https://www.mdpi.com/article/10.3390/pathogens14080728/s1, References [84,85,86,87,88,89,90,91,92,93,94,95,96,97,98,99,100,101,102,103,104,105,106,107,108,109,110,111,112,113,114,115,116] are cited in the Supplementary Materials. Table S1: Descriptive summary of reviewed studies on canine rabies control and community KAP. Table S2: Summary of knowledge, attitudes, and practices (KAP) on rabies and responsible dog ownership reported across reviewed articles. Table S3: The reasons for owners failing to vaccinate their dogs. Figure S1: The relationship between vaccination coverage and the proportion of dog owners providing evidence of vaccine records (n = 12). The dotted trendline indicates a positive association between documented vaccination and reported vaccination coverage (ρ = 0.78, *p* = 0.002). Figure S2: Scatter plot showing the relationship between ranked belief that vaccination is useful and ranked dog vaccination practices (n = 15). The dotted trendline indicates a positive correlation (ρ = 0.69, *p* = 0.004). Figure S3: Scatter plot illustrating the relationship between ranked awareness that rabies is fatal and ranked dog vaccination practices (n = 11). The dotted trendline indicates a weak positive and non-significant correlation (ρ = 0.25, *p* = 0.47). Figure S4: Scatter plot showing the relationship between ranked dog roaming behavior and ranked dog vaccination practices (n = 17). The dotted trendline indicates a negative but non-significant correlation (ρ = −0.36, *p* = 0.16).

## Abbreviations

The following abbreviations are used in this manuscript:

PEP	Post-Exposure Prophylaxis
WHO	World Health Organization
PAHO	Pan American Health Organization
KAP	Knowledge, Attitudes, and Practices
RDO	Responsible Dog Ownership
PRISMA	Preferred Reporting Items for Systematic Reviews and Meta-Analyses
MeSH	Medical Subject Heading
ORV	Oral Rabies Vaccination
