# Supplementary material for: Global Perspectives on Rabies Control and Elimination: A Scoping Review of Dog Owners’ Knowledge, Attitudes, and Practices
Source: Pathogens. 2025 Jul 23;14(8):728. doi: 10.3390/pathogens14080728 (PMC12389161; doi:10.3390/pathogens14080728)
Supplement: Supplementary file 1 [file pathogens-14-00728-s001.zip › pathogens-3735708-supplementary.pdf]

Table S1. Descriptive summary of reviewed studies on dog rabies control and community KAP

Authors	Year	Sample size	Country	Journal name	Key study findings
Agustina et al. [78]	2017	200 households (52.2% dog-owning)	Indonesia	Jurnal Veteriner	Participants showed a high level of knowledge about rabies risk and vaccination, but fewer than one-third were aware of preventive measures.
Ahmed et al. [80]	2022	326 dog-owning households	Ethiopia	Journal of Public Health Research	More than half (52.1%) of participants exhibited inadequate practices regarding rabies prevention and control.
Ameh et al. [79]	2014	200 dog owners	Nigeria	Global Journal of Health Science	Only 13% of respondents knew that the rabies virus is found in nervous tissue, and 86% were unaware of the appropriate age for rabies vaccination.
Amemiya et al. [84]	2023	534 dog owners	Japan	Vaccines	A statistically significant association was found between dog rabies vaccination and several factors, including owner education level, mandatory vaccination policy, access to a family veterinary clinic, frequency of veterinary visits, and prior advice to vaccinate.
Awoyomi et al. [62]	2019	268 dog owners	Nigeria	Nigerian Veterinary Journal	Dog rabies vaccination coverage was low (4.9%). Inadequate knowledge about dog vaccination was significantly associated with age, education level, and occupation.
Bahiru et al. [85]	2022	209 dog owners (from	Ethiopia	Heliyon	Approximately 61% of respondents had

		899 households)			sufficient knowledge, 72% demonstrated a favorable attitude, and 45% achieved good practice scores regarding rabies control.
Bardosh et al. [63]	2014	113 dog owners	Tanzania	Plos Neglected Tropical Diseases	Only 25% of dogs were reported to be vaccinated across the six sampled villages.
Bihon et al. [81]	2020	163 dog owners (out of 384 respondents)	Northwest Ethiopia	Diseases (MDPI)	Overall, 51% of participants exhibited poor knowledge, attitudes, and practices regarding rabies.
Bitsu Kiflu et al. [77]	2016	252 dog-owning households	Ethiopia	Ethiopian Veterinary Journal	About 87% of respondents believed that dogs could transmit zoonotic diseases, and 95.4% were aware of rabies.
Bouaddi et al. [82]	2020	380 dog owners (out of 407 respondents)	Morocco	Veterinary Sciences (MDPI)	Although most respondents were aware of the benefits of vaccination, many did not vaccinate their dogs and instead relied on traditional healers for rabies treatment.
Bundalian et al. [40]	2020	425 dog owners (out of 453 respondents)	Philippines	National Public Health Journal	While knowledge of responsible dog ownership (RDO) was generally good, the study recommended further education for dog owners.
Chaudhary et al. [86]	2018	222 dog owners (out of 459 respondents)	Nepal	Journal of Kasetsart Veterinarians	Pet owners were found to be more knowledgeable about rabies. The study recommended increasing rabies awareness and conducting mass vaccination campaigns.

Chikoondo et al. [87]	2019	100 dog owners	Tanzania	Tanzania Veterinary Journal	Low vaccination coverage was attributed to poor knowledge about rabies and misconceptions regarding vaccination campaigns.
Christopher et al. [35]	2021	53 dog owners (out of 175 respondents)	Indonesia	International Maritime Health	The study identified significant knowledge gaps regarding rabies and dog population management.
Costa et al. [88]	2018	83 dog owners (out of 208 respondents)	Cameroon	PLOS ONE	The majority of dogs were reported to be free-roaming and unvaccinated.
Dahourou et al. [67]	2021	316 dog-owning households	Burkina Faso	Veterinary and Animal Science	People with prior rabies knowledge were more likely to vaccinate their dogs; association was statistically significant.
Daigle et al. [89]	2023	47 dog owners (out of 122 respondents)	Northern Quebec, Canada	Frontiers in Veterinary Science	Lack of awareness about dog bite and rabies risk found in an Indigenous Canadian community.
Das et al. [36]	2025	33 veterinary professionals	Turkana County, Kenya	Frontiers in Veterinary Science	Less than half of the population was considered knowledgeable about rabies or dog vaccination, with key barriers including poor awareness, lack of campaign information, and vaccination costs
Davlin et al. [90]	2014	351 dog-owning households (out of 460)	Philippines	Epidemiology and Infection	Participants with good rabies knowledge were willing to vaccinate and register their dogs.
Dhakal et al. [91]	2023	70 pet owners (out of 308 respondents)	Nepal	International Journal of Environmental Research and Public Health	Dependence on traditional healers for rabies treatment persists; mass education is needed.

Dizon et al. [51]	2022	393 dog-owning households (out of 727)	Philippines	PLOS Neglected Tropical Diseases	General rabies awareness was good, but significant gaps remained in transmission knowledge.
Ebuy et al. [45]	2019	721 dog owners (out of 1440 respondents)	Ethiopia	Nigerian Veterinary Journal	72.2% had good knowledge, 66.0% good attitude, and 62.4% good practices regarding rabies.
Edukugho et al. [33]	2018	123 dog owners (out of 224 respondents)	Nigeria	Pan African Medical Journal	Dog owners were 7.8% more knowledgeable about rabies than non-dog owners; gaps existed in vaccination schedule, roaming restriction, and PEP.
Eshetu Yimer et al. [76]	2012	969 dog-owning households (out of 2390)	Ethiopia	Ethiopian Veterinary Journal	Very poor responsible dog ownership (RDO) practices reported, including feeding, housing, healthcare, and vaccination.
Fielding et al. [92]	2012	744 dog-owning households (out of 1290)	Haiti	Journal of Applied Animal Welfare Science	Strong recommendation for public education on RDO, including vaccination, neutering, population control, and welfare.
Glasgow et al. [93]	2019	617 animal-owning households (out of 996)	Granada	PLOS Neglected Tropical Diseases	Participants lacked awareness of past vaccination campaigns and had limited knowledge about rabies-risk animals.
Hasanov et al. [57]	2021	100 dog and livestock owners	Azerbaijan	PLOS ONE	Most participants had heard of rabies but lacked knowledge about preventive measures and transmission routes.
Hiby et al. [94]	2018	2594 dog owners (out of 3171 respondents)	Indonesia	Animals (MDPI)	High dog turnover challenged rabies control; abandoned and young dogs were at

					higher risk of being unvaccinated.
Hudson et al. [95]	2016	31 dog owners	Australia	PLOS Neglected Tropical Diseases	Limited veterinary service contributed to low vaccination rates; some owners resisted dog movement restrictions.
Iddi et al. [96]	2023	422 dog owners	Tanzania	PLOS Neglected Tropical Diseases	Although 90% supported vaccination, only half vaccinated their dogs; higher education and farming background linked to positive attitudes.
Ijoma et al. [34]	2021	66 dog owners	Nigeria	Nigerian Veterinary Journal	Very poor rabies knowledge was reported among dog owners.
Ishola et al. [97]	2021	138 dog owners	Nigeria	Veterinaria Italiana	Despite awareness of rabies, vaccinated dogs had low immunity; improved vaccination strategies recommended.
Jama and Mengistu [32]	2023	49 dog owners (out of 384 respondents)	Ethiopia	Journal of Zoonotic Diseases	Good overall KAP score, but lacked advanced knowledge on rabies biology and PEP.
Javed et al. [98]	2023	1686 pet owners (out of 2000 respondents)	Pakistan	International Journal of Veterinary Science	Basic rabies knowledge was common, but deeper understanding and bite management practices were lacking.
Kadowaki et al. [43]	2018	495 dog-owning households	Vietnam	Preventive Veterinary Medicine	Low rabies awareness among low-income and mountain ethnic groups; local language outreach recommended.
Khan et al. [99]	2019	214 dog owners (out of 434 respondents)	Pakistan	Acta Tropica	Poor KAP scores observed; mass education and awareness campaigns advised.

Konzing et al. [80]	2021	100 dog owners	Nigeria	Nigerian Veterinary Journal	Overall KAP scores were satisfactory, but most respondents were unaware that rabies is fatal.
Leblanc et al. [100]	2024	370 participants from both the quantitative + qualitative survey approaches	Moramanga district, Madagascar	PLoS Neglected Tropical Diseases	While general knowledge about rabies transmission, symptoms, and outcomes was relatively high in Madagascar, awareness of treatment centers, post-exposure prophylaxis, and proper response to dog bites was very low, especially in remote rural areas.
Lunney et al. [83]	2012	141 dog-owning households (out of 250)	Cambodia	International Health	Most dog owners showed a positive attitude toward vaccination, though few were interested in neutering or spaying.
Mapatse et al. [46]	2022	233 households (90% owned at least one dog)	Mozambique	PLOS Neglected Tropical Diseases	Poor rabies knowledge and practices linked to low education and lack of modern infrastructure.
Massei et al. [101]	2017	60 dog owners	Nepal	Zoonoses and Public Health	Reported poor knowledge about PEP and RDO; recommended mass education on first aid following bite exposure.
Mauti et al. [81]	2017	279 dog-owning households (with 379 dogs)	Mali	Acta Tropica	Low dog vaccination coverage; suggested repeated annual vaccination campaigns including puppies under 3 months old.
Mbilo et al. [38]	2021	45% of 963 households owned at least one dog	Chad	Acta Tropica	Majority had poor knowledge about rabies, PEP, and other preventive measures.

Mbilo et al. [60]	2019	100 dog-owning households (out of 1056)	Congo	Vaccine (MDPI)	Rabies-related human deaths reported; low public awareness identified as a barrier.
Menghistu et al. [102]	2018	Mekelle: 252, Asella: 146, Kisumu: 183, Siaya: 168	Ethiopia & Kenya	Ethiopian Journal of Health Development	High number of free-roaming dogs and bite incidents; rabies control hindered by poor awareness and ineffective population management.
Mohammad et al. [28]	2025	32 dog owners	The North-Eastern Region of Peninsular Malaysia	One Health Outlook	Just over half of dog owners had good knowledge of rabies, while positive attitudes (26.7%) and good practices (40%) were notably lower.
Nejash and Endale [103]	2017	67 dog owners (out of 150 respondents)	Southern Ethiopia	Journal of Public Health and Epidemiology	57% had a satisfactory KAP score, but advanced rabies knowledge was lacking; mass awareness programs recommended.
Njoga et al. [104]	2020	309 dog owners	Nigeria	Animal Research International	Few respondents knew about contraceptive methods like castration, movement restriction, pills, or hormone injections for rabies control.
Ntampaka et al. [53]	2019	137 dog owners	Rwanda	PLOS ONE	Respondents showed good KAP on rabies, but improvements in prevention and treatment practices were still needed.
Omar et al. [105]	2023	600 respondents including 142 dog owners	Tanzania	Tropical Medicine and Infectious Disease	Dog collars used during mass vaccination campaigns positively influenced community perceptions and behaviors. 57% of respondents felt safer around collared dogs, and 64% of dog owners

					were more likely to vaccinate their dogs because of the collars.
Premashthira et al. [54]	2021	476 dog owners	Thailand	Frontiers in Veterinary Science	Younger male participants having a middle to high school education, or owned more dogs tended to possess good knowledge about rabies prevention but showed less attitudes. Lower-income farmers exhibited more positive attitudes regardless of their level of knowledge.
Remedios D. San Jose et al. [41]	2020	336 dog owners (out of 380 respondents)	Philippines	Philippine Journal of Veterinary Medicine	Most dog owners practiced responsible ownership, including vaccination, leashing, feeding, and restricting dog movement.
Sambo et al. [55]	2014	17% dog-owning households (out of 5141)	Tanzania	PLOS Neglected Tropical Diseases	Only half vaccinated their dogs; contributing factors included male-headed households, large family size, low income, urban location, and livestock ownership.
Sikana et al. [106]	2021	66 dog owners (out of 417 households)	Tanzania	PLOS Neglected Tropical Diseases	Vaccination decisions were male-dominated; 83% of dogs were reportedly vaccinated.
Sor et al. [37]	2018	360 dog owners	Cambodia	Tropical Medicine and Health	Dog vaccination coverage was low due to high dog turnover, lack of awareness, and poor dog population control.
Spargo et al. [107]	2021	395 pet owners (out of 798 respondents)	Zimbabwe	PLOS ONE	Pet owners and healthcare professionals were significantly more knowledgeable about

					rabies than other respondents.
Srinivasan et al. [39]	2021	37 pet owners (out of 130 respondents)	India	National Journal of Community Medicine	Over 90% supported dog vaccination; 70% had good knowledge of PEP.
Swacita et al. [108]	2023	74 dog owners (out of 234 respondents)	Indonesia	International Journal of Veterinary Science	Over 80% supported vaccination and sterilization as rabies control strategies, especially in high-risk areas.
Tapdasan et al. [42]	2017	150 dog owners (out of 400 respondents)	Philippines	Philippine Journal of Veterinary Medicine	Although participants were concerned about rabies and RDO, they lagged in practicing vaccination, leashing, registration, and neutering.
Tenzin et al. [109]	2024	More than 3,700 households	The Northern Communal Areas of Namibia	PLOS Neglected Tropical Diseases	While general awareness and treatment-seeking behavior after dog bites were strong, gaps in dog vaccination, post-bite prophylaxis, and rabies surveillance persist.
Thomas et al. [68]	2013	120 dog owners	Grenada	Preventive Veterinary Medicine	All respondents supported vaccination as the best rabies control method, but actual coverage was low.
Ubeyratne et al. [44]	2020	780 dog owners	Sri Lanka	Transboundary and Emerging Diseases	Although 69% saw dogs as rabies reservoirs, many didn't restrict dogs indoors; PEP uptake was relatively high.
Ung et al. [110]	2021	310 dog owners	Cambodia	Veterinary World	Overall KAP was unsatisfactory; about half did not vaccinate their dogs but showed willingness if the vaccine was free.

Velander et al. [50]	2022	359 dog owners	Laos	Pathogens	24% of unvaccinated dogs tested ELISA positive, possibly due to contact with rabid dogs or unauthorized immunization.
Voupawoe et al. [111]	2022	1158 dog-owning households (out of 1282)	Liberia	Acta Tropica	Rabies knowledge was very low; male adults were the most common bite victims.
Wallace et al. [112]	2017	12.9% dog-owning households (out of 798)	Uganda	Infectious Diseases of Poverty	Vaccination coverage was low; dog ownership was linked to lower economic status and densely populated areas.
Wera et al. [61]	2015	450 dog owners	Indonesia	PLOS Neglected Tropical Diseases	Half of respondents vaccinated their dogs; lack of awareness about vaccination campaigns was the main barrier.
Wera et al. [113]	2016	450 dog owners	Indonesia	Preventive Veterinary Medicine	Respondents favored culling for rabies control; willingness to vaccinate decreased significantly when vaccination wasn't free.
Widyastuti et al. [73]	2015	72% dog owners (out of 300 respondents)	Indonesia	Infectious Diseases of Poverty	Community had fair knowledge and positive attitude toward rabies control, but vaccination rates were low.
Wolelaw et al. [64]	2022	523 dog owners (out of 609 respondents)	North West Ethiopia	Scientific Reports	Rabies prevention practices were low and were strongly associated with knowledge, attitude, and other demographic factors.

Table S2: Summary of Knowledge, Attitudes, and Practices (KAP) on rabies and responsible dog ownership reported across reviewed articles

KAP	Parameter	No. of articles	References
Basic understanding of rabies			
Knowledge	Heard about rabies	26	[35,36,40,42,45,46,50,51,55,57,60,73,77,78,80,83,90,91,98–100,102,103,107,110,111]
	Dogs can act as a reservoir of rabies	18	[32,35–37,39,44,46,76,77,79,82,85,96,99,100,107,111]
	Identify clinical signs of rabies	7	[28,33,34,36,67,99,107]
	Rabies has no cure once clinical signs appear	20	[32,35–38,40,44–46,50,60,61,67,80,87,98,100,103,110,111]
Vaccination of dogs			
Knowledge	Rabies vaccination is useful for dogs	29	[28,32,36–38,42,46,51,53,55,57,60,68,78,80–82,86,93,96,99,100,102,103,107,110,111,113]
	Know the rabies vaccination schedule for dogs	7	[35,39,62,67,80,81,85]
	Rabies vaccination should be repeated annually	5	[33,36,73,79,82]
Attitude	Owner's willingness to vaccinate (free of cost)	5	[43,57,62,110,113]
	Owner's willingness to pay for vaccination	11	[41,43,45,57,60,62,81,83,90,110,113]

Practice	Perform vaccination of dogs	47	[32–34,36–45,50,53,55,60,61,63,64,67,76,78,79,81,84,85,87,88,90–95,97,98,100,101,104–106,108,109,112]
	Keeping records/evidence of vaccination	12	[33,34,38,40,41,45,67,68,78,90,104,112]
Responsible dog ownership			
Knowledge	Leashing is important	3	[40–42]
	Sterilization/neutering can control rabies	2	[40,42]
	Providing food, water, and shelter	1	[41]
	Dog registration and licensing	5	[40,42,79,80,82]
	Regular veterinary care	2	[41,107]
Attitude	Maintain leashing	5	[35,41,43,73,113]
	Sterilization/neutering can control rabies	3	[41,83,108]
	Providing food, water, and shelter	1	[44]
	Dog registration and licensing	3	[32,45,46]
Practice	Maintain leashing	12	[28,37,41,42,55,64,73,78,84,87,91,108]

	Allowed dogs to roam outside	24	[32,33,40,46,50,55,60,64,73,76–79,86–88,91,94–96,108,112,114,115]
	Sterilization/neutering	8	[42,46,51,61,78,86,95,104]
	Providing food, water, and shelter	10	[41,42,55,67,76,77,84,88,101,112]
	Dog registration and licensing	2	[32,84]
	Regular veterinary care	7	[41,46,60,67,80,88,112]

Table S3: The reasons for owners failing to vaccinate their dogs

Factors	Components	Number of publications	References
knowledge-related barriers or misperception	Regarding rabies and the vaccination campaign, government-supported campaigns, the risk of exposure to other diseases during vaccination campaigns	12	[36,38,46,53,60–64,68,81,112]
Issues with the Vaccine	High price, vaccine availability, accessibility of vaccine providers/veterinarians, side effects, owner's belief in vaccine's effectiveness	12	[36,38,50,53,61–64,67,81,112,116]
Distance to Clinic	Distance to the veterinary clinic, lack of transportation	7	[36,38,50,62,67,68,81]
Personal Constraints of Owners	Negligence, inability to handle dogs, young age of owned dogs, dogs left outside during campaign, recent relocation, refusal to disclose reasons	10	[36,38,46,53,60,61,63,67,68,81]

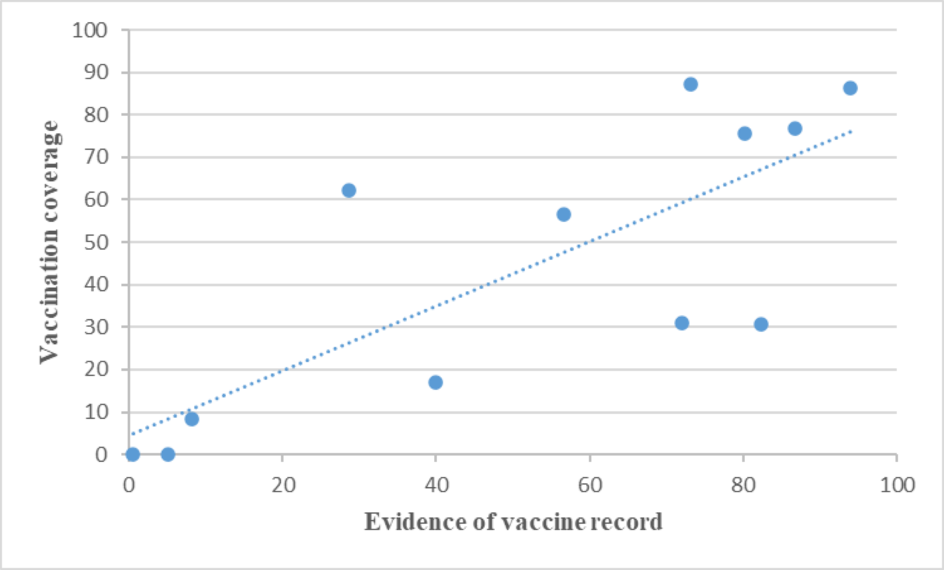

Figure S1. The relationship between vaccination coverage and the proportion of dog owners providing evidence of vaccine records (n = 12). The dotted trendline indicates a positive association between documented vaccination and reported vaccination coverage (ϱ = 0.78, p = 0.002).

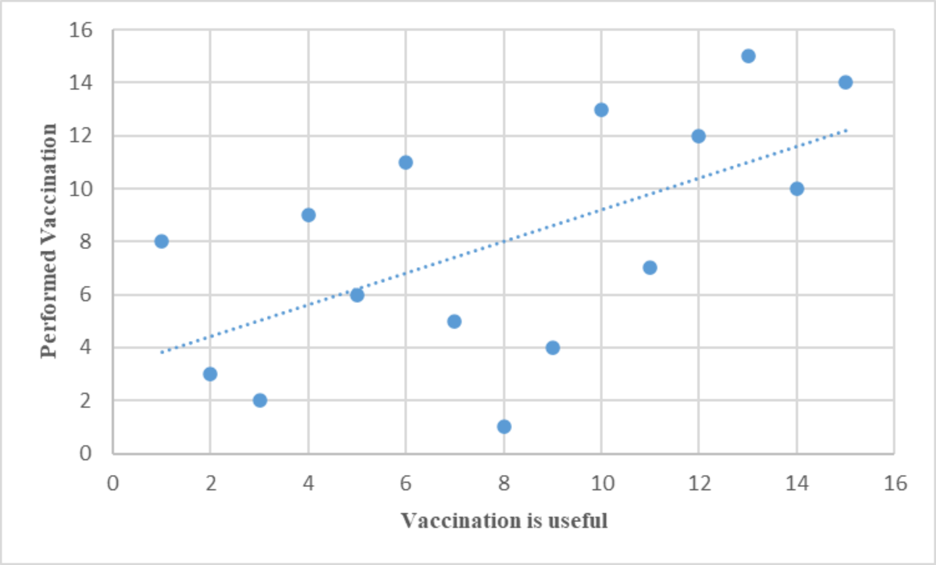

Figure S2: Scatter plot showing the relationship between ranked belief that vaccination is useful and ranked dog vaccination practices (n = 15). The dotted trendline indicates a positive correlation (ϱ = 0.69, p = 0.004).

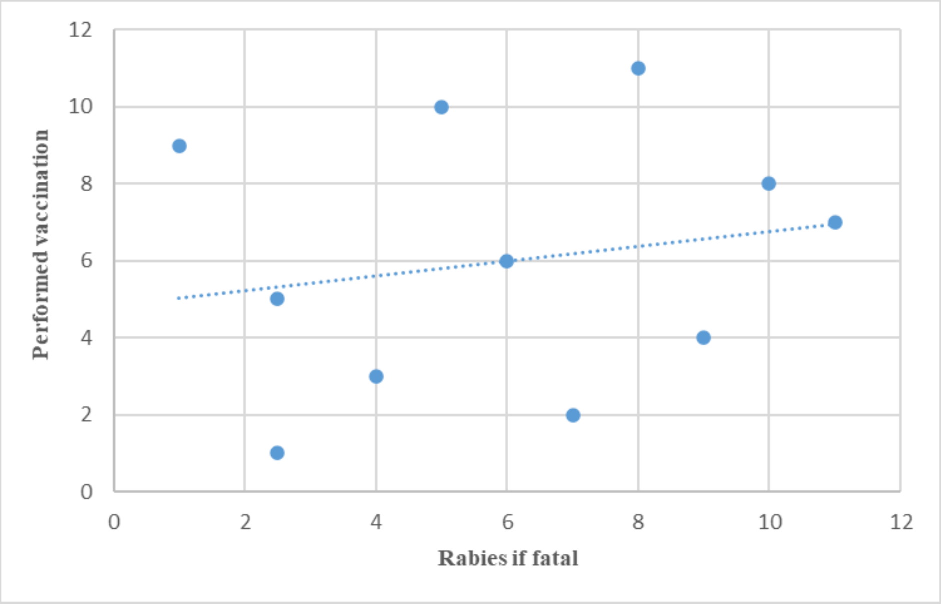

Figure S3: Scatter plot illustrating the relationship between ranked awareness that rabies is fatal and ranked dog vaccination practices (n = 11). The dotted trendline indicates a weak positive and non-significant correlation (ϱ = 0.25, p = 0.47).

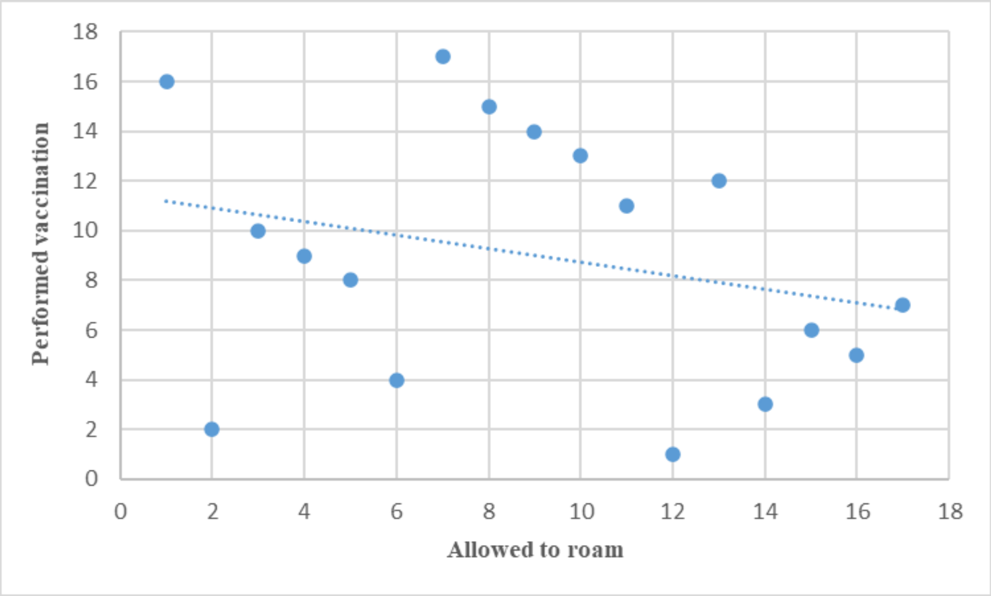

Figure S4: Scatter plot showing the relationship between ranked dog roaming behavior and ranked dog vaccination practices (n = 17). The dotted trendline indicates a negative but non-significant correlation (ϱ = −0.36, p = 0.16).
